# Exploring healthcare workers’ perceptions on the use of morbidity and mortality audits as an avenue for learning and care improvement in Kenyan hospitals’ newborn units

**DOI:** 10.1186/s12913-022-07572-8

**Published:** 2022-02-10

**Authors:** Joyline Jepkosgei, Jacinta Nzinga, Mary B. Adam, Mike English

**Affiliations:** 1 Health Services Unit, KEMRI-Wellcome Trust Research Programme, P. O. Box 43640 – 00100, 197 Lenana Place, Lenana Road, Nairobi, Kenya; 2 AIC Kijabe Mission Hospital, Kijabe, Kenya; 3 Centre for Tropical Medicine and Global Health, Nuffield Department of Medicine, University of Oxford, Oxford, UK

**Keywords:** Newborn, Perinatal, Maternal, Morbidity, Mortality, Audit

## Abstract

**Background:**

In many sub-Saharan African countries, including Kenya, the use of mortality and morbidity audits in maternal and perinatal/neonatal care as an avenue for learning and improving care delivery is sub-optimal due to structural, organizational, and human barriers. While attempts to address these barriers have been reported, lots of emphasis has been paid to addressing the role of tangible inputs (e.g., availing guidelines and training staff in the success of mortality and morbidity audits), while process-related factors (i.e., the role of the people, their experiences, relationships, and motivations) remain inadequately explored. We examined the processes of neonatal audits, their potential in promoting learning from gaps in care and improving care delivery, with a deliberate focus on process-related factors that generally influence mortality and morbidity (M&M) audits.

**Methods:**

This was an exploratory qualitative study, conducted in three hospitals, in Nairobi and Muranga counties. We employed a mix of in-depth interviews (17) and observation of 12 mortality and morbidity audit meetings. Our study participants included: nurses, doctors, trainee clinicians (i.e., junior doctors on internships), and nursing students involved in providing newborn care. These data were coded using NVivo12 employing a thematic content analysis approach.

**Results:**

Perceived shortcomings in the conduct of M&M audits such as unclear structure was reported to have contributed to its sub-optimal nature in promoting learning. These shortcomings, in addition to hierarchy and power dynamics, poor implementation of audit recommendations, and negative experiences, (e.g., blame) also demotivated health workers from attendance and participation in audits. Despite these, positive outcomes linked to audit recommendations, such as revision of care protocols, were reported. Overall, leadership and a blame-free culture enabled positive changes and promoted learning from audit-identified modifiable factors.

**Conclusion:**

Our findings indicate that M&M audits provide a space for meaningful discussions, which may lead to learning and improvement in care delivery processes. However, a lack of participation, lack of observed positive outcomes, and negative experiences may reduce their usefulness. An enabling environment characterized by minimized effects of hierarchy and positive use of power and a blame-free culture may promote active participation, enhancing positive relationships and interactions thus promoting team learning.

**Supplementary Information:**

The online version contains supplementary material available at 10.1186/s12913-022-07572-8.

## Background

Learning from errors and gaps that compromise the quality of care by healthcare teams, provides an opportunity for addressing team and institutional level failures, which could range from obvious technical errors such as wrong-site surgery to invisible breakdowns in communication such as a nurse’s failure to challenge a physician’s questionable medication order, and thus improving patient safety [1]. Edmonson argues that learning at the institutional level is the cumulative result of learning among small groups or small health care teams at the unit/patient care level [2]. For healthcare teams to achieve learning, they must continuously engage in local learning processes [3, 4]. These processes, which include multidisciplinary ward rounds and morbidity and mortality (M&M) audits, enable teams to identify errors and gaps in care, reflect on these and develop action points to prevent future recurrence [5–7].

M&M audits have increasingly been used in hospitals both in High-Income Countries (HICs) and Low-and Middle-Income Countries (LMICs) [8–10], as a tool for learning from failures to consequently improve the quality and safety of patient care [11]. Traditionally, M&M audits have been used by clinical teams to review cases that may have ended in an adverse event or mortality [12–15]. M&M audits aim to identity correctable deficiencies (e.g. medical errors) such as omission in care, delayed or missed diagnosis, inappropriate or missed drug administration, miscommunication, and where necessary, take immediate steps to improve care. When done routinely, facility-based morbidity and mortality audits have been shown to improve the quality of care and patient outcomes [8, 9]. To achieve these, audit teams must operate in an environment that fosters trust and fairness, commonly known as “just culture” [16, 17]. This culture promotes accountability, specifically, the ability of health workers to openly report gaps in care/disclose errors, to take responsibility where actions are within their control, and to seek help and feedback from each other and the hospital management where required, without fear of being blamed or victimized [18, 19]. Finally, team learning must be spearheaded and inspired by dedicated learning-oriented team leadership that is visible and accessible to all [11, 17].

International guidelines recommend that mortality audits should be carried out by multi-disciplinary audit teams, whose members meet regularly to review events of a case that ended in mortality [20]. These guidelines further suggest that a structured format is adopted, to aid in the gathering of evidence and evaluation of whether (and how) current hospital practices contributed to the death. Audit team members should engage in discussions about what went well, what didn’t, what to do differently the next time, and whether it was an inevitable death (that is, a medically indicated outcome). The audit team then discusses their findings to identify correctable deficiencies and, where warranted, take immediate steps to put new procedures in place [21, 22].

In LMICs, mortality audits have gained traction in maternal care since the early 1990s [23]. More recently, a call to explore the underlying causes of stillbirths and neonatal deaths, and avoidable factors recommend integrating routine perinatal death reviews/audits into existing systems [24]. In response, most LMICs have adopted national guidelines that modified maternal audits/death reviews audits to include perinatal audits. A recent review indicates that maternal and perinatal morbidity and mortality reviews are often not done [25]. At the time of the review (2019), while several sub-Saharan African countries had initiated the procedures for the conduct of maternal and perinatal death and surveillance response (MPDSR) audits, only a few had these audits happening at a national scale. The minimal implementation progress was further undermined by weak structural and organizational capacities [17, 26–28]. For example, in Kenya, progress with implementation has been slow, in part due to the low number of notifications and deaths reported, poor completion of death review forms, lack of data use, lack of evidence of response to data findings, and fear of being blamed for poor outcomes [27].

While recommendations have been made to address existing challenges to the successful implementation of audits, the influence of process-related factors, particularly the role of the people, their interactions and relationships, motivations, and their communication mechanisms on the implementation process remains largely unexplored [26]. In this qualitative study, we sought to explore the structure (how meetings are organized: participants, frequency and presentation of cases) and processes of newborn M&M audits and the perceptions of audit team members on the use of audits as a space for learning from gaps in care, and care improvement. In doing this, we deliberately explored the process-related factors that generally influence M&M audits in health care including professional interactions and their experiences, institutional cultures, and broader health system contextual influences.

## Methods

### Study design

This qualitative exploratory study utilized an ethnographic approach in which non-participant observations, and interviews [29] with health care providers were carried out. It is part of a broader study exploring intra- and interprofessional teamwork and team processes of care providers working in the NBUs. In this study, audits and care provision processes, including ward rounds, handovers, and departmental meetings were selected as tracer activities for interprofessional teamwork and team interactions based on our past NBU ethnography work [30, 31]. This paper mainly draws on audit observation data and interviews, where we also explored M&M audit structure and processes, and how these facilitated team learning.

### Study sites

The study was conducted in three hospitals located in two counties in Kenya. These hospitals were purposively selected to maximise diversity across the following characteristics: situated in rural and urban settings; level of neonatal services offered (including differences in the number of in-patient admissions) and type of ownership (including public and faith-based). Across the study hospitals, NBUs are staffed by nurses; clinical/medical officers; pediatrician/a neonatologists; and trainee clinicians. Of these, the nurses are the most numerous care providers across NBU work shifts.

### Study sample

To understand the audit process and to observe interactions/learning during audit meetings, the first author (JJ) attended and observed 12 audit sessions across the three hospitals: 5 in hospital X, 2 in hospital Y (during the data collection period, the NBU department only managed to conduct two audits) and 5 in hospital Z. The audit team members varied across hospitals with attendees mostly comprised of nurses, medical officers, and medical and clinical officer interns drawn from various departments. Nurses, doctors, and trainee clinicians were purposively selected for in-depth interviews by considering years of working experience in the unit, managerial position, and training level.

### Data collection and analysis

A semi-structured observation guide was developed and used to direct the observations during all the audit sessions. Some of the key aspects explored during these observations were drawn from literature, which included: composition of the audit team; roles of audit team leaders; nature of interactions; professionalism (the existence (or not) of collective decision making, respectful exchange of ideas/opinions and reference to existing guidelines); and the agency of team members (the dynamics of participation by different professions, considering their seniority levels, and how team members discussed and solved arising safety concerns/gaps in care). A more detailed observation guide is provided (see Additional file 1). JJ took field notes that were later typed in MS word, coded on NVivo together with interview transcripts, and incorporated in the analysis.

Insights from the observation of audit sessions were used to develop the interview guide. This interview guide was pilot tested; however, changes were also made iteratively as interviews progressed. The final version of the guide is provided (see Additional file 2). All interviewees were invited for interviews, which were conducted with participants at the workplace. During the interviews, only the participant and the researcher were present. All the interviews lasted about 1 h, were conducted in English by JJ and were audio-recorded with consent from the participants. The interviews explored audit team members’ experiences of M&M audit processes, their knowledge of existing guidelines and their use, and overall, the perceived positive changes observed as a result of conducting audits.

The field notes and interview transcripts were then imported to NVivo 12 qualitative research software as a shared project. We adopted a thematic content analysis approach, which involved: data familiarization through reading and re-reading the transcripts; generating initial codes; grouping these codes to broader descriptive themes by matching patterns and relating these to existing literature. In the first phase of the analysis, two authors, JJ and JN, independently coded the data. This was then followed by intensive discussions between the two authors; who made comparisons between the individually identified codes and based on this, a consensus was reached on the coding framework. All data were then coded, guided by the coding framework using NVivo 12. However, this was an iterative and flexible process, and the coding framework was updated as the data coding progressed allowing for emerging codes. The coded data was drawn to support the categorization and presentation of the emerging themes, which are presented in the Results section below.

## Results

In total, 12 audit sessions, each lasting 2 h on average, were observed, and a total of 17 interviews were conducted with staff across all hospitals. Table 1 provides a summary of study hospital characteristics and the participants sampled per hospital.

**Table 1:** Study hospital characteristics & study sample summary

Characteristics	Description		
Hospital code	X	Y	Z
No of admissions	38 admissions monthly (456 yearly)	27 admissions monthly (324 yearly)	90 admissions monthly (1080 yearly)
Audit type	Maternal/perinatal/neonatal	Neonatal	Neonatal
Frequency	Monthly	Monthly	Monthly
In-depth interviews (17)	Nurses – 2 2 Nursing students – (1interview) 3 Medical officer interns – (1 interview) Pediatrician – 1	Nurses – 3 Clinical officer – 1 Pediatrician 1	Nurses – 4 Clinical officer – 1 Medical officer – 1 Neonatologist – 1
Number of audit meetings observed	5	2	5

### Emerging themes

In this section, we describe five broad emerging themes: the structure of M&M audits, audit team members’ experiences of the audit process, perceived outcomes of M&M audits, the perceived role of leadership in promoting successful audits and the role of culture in enabling audits as a potential space for promoting learning and being accountable for mistakes to continuously improve care delivery processes.

### Structure of morbidity and mortality meetings

We describe the M&M audit meeting structure below in three sub-themes; who are involved, audit frequency, and the case review process. A summary of audit structure and process characteristics is provided (see Additional file 3).

### Participation

Across the study hospitals, M&M meetings are attended by various frontline healthcare providers including medical and clinical officers^1^ [32]; medical clinical and officer interns; nurse managers, and consultants: either a paediatrician, gynaecologist or neonatologist. Despite their central role in caring for newborns, nurses and nursing students were often missing or inconsistently attended audit meetings. Their absence was partly explained by work constraints due to staff shortages, and strongly held notions that M&M audit meetings were a doctor-oriented learning activity. While nurse managers regularly attended the meetings on behalf of frontline nurses, the absence of other nursing staff greatly compromised the potential for interprofessional learning from case reviews.

In one study hospital, participants included representatives from multiple units within the hospital and peripheral facilities (i.e., sub-county health centres); meeting deliberations on cases were exhaustive. We observed that such broad participation allowed for consensus and more meaningful action planning across departments and the various levels of care. For example, in one facility where only newborn unit (NBU) audits were conducted, we observed that often, the maternity unit was pointed out as the source of some of the identified gaps in care. However, representatives from this unit were absent in the meetings, and in such instances, there appeared to be no opportunities for learning from mistakes. Consequently, addressing the gaps at NBU only did not imply that these scenarios would not recur.

### Frequency of M&M meetings

M&M audit meetings were conducted monthly across all study hospitals. However, in one hospital, in addition to the meetings, audits would be convened by a smaller team in the event of a poor outcome, for instance, sudden change in a patient’s condition that may require critical care. These immediate review meetings allowed care providers to quickly brainstorm and identify correctable deficiencies, leading to timely action and response to the situation.

### Case review process

Across the study hospitals, junior doctors and trainee clinicians were tasked with selecting the cases for review, reviewing these cases, and providing a summary presentation of the cases during audit meetings. Only hospital Y had adopted a structured localized approach to case reviews. There, the audit team used a fishbone diagram or Ishikawa (a visualization tool used to analyze potential causes of a problem/process failures) [33] to identify errors and gaps in care that could be either linked to: the people providing care, inappropriate procedures, existing policies, equipment, or the clinical environment. From our observations, this approach encouraged detailed identification of modifiable deficiencies and gaps in care, following which appropriate action points were drafted and agreed on. This approach seemed to enable team learning and subsequent implementation of recommended actions by incorporating these in routine practice.

In the two other study hospitals, the review process was unstructured. Select presenters (often the doctors) provided a summary of the case and brief discussions on the cases are conducted with rapporteurs (mostly doctors), taking note of meeting deliberations and recommended action points. However, we observed hospitals X and Z lacked a mechanism for follow-up on action points and therefore, subsequent meetings were laden with a recurrence of similar concerns, derailing the potential of the use of M&M audits as spaces to promote learning. Consequently, healthcare providers demonstrated a lack of interest in audit meeting attendance. In one hospital, sub-optimal participation by all frontline healthcare providers was suggested to compromise the effectiveness of M&M audits in addressing gaps in care.

### Audit team members’ experiences of the audit process

Across the study hospitals, health workers reported varied experiences of attending audit meetings. Some clinicians perceived M&M as a useful avenue for learning, although often sub-optimal and not able to exhaustively identify gaps in care throughout the care pathway.

*Yes, it is useful, yes but now like there is a time we used to have audits- 30 minutes case presentation. That is never done so we come to audits they extend, extend people get bored people to start leaving… so they should be planned actually, be time conscious…(Clinical officer) [hospital Z]*

Furthermore, participants often found the structure of audits (i.e., how the case review process is structured) to be unclear which may have contributed to the sub-optimal nature of audit meetings, by hindering an elaborate identification of gaps in care throughout the care pathway. This suggests that some of the case review styles adopted, for example in hospital Z, is not useful for adequately and logically identifying deficiencies in care delivery that can inform action points.

*Yes, for me I would say there is just… it is not optimal because I have also attended other forms of audits that analyze from at the point of admission, what happened, At the point of referral, admission, delivery, post-delivery going all the way to post-mortem. And then now looking at the various parts of care that the delays, looking at the delays and the post-natal care I know that is the way it should happen. (Clinical officer) [hospital Z]*

Furthermore, we observed minimal participation among some health care providers. These could have been explained by power displays over others and often along professional hierarchies, for example, in hospital Z, sitting arrangements during audit meetings accentuated power dynamics, limiting interprofessional interactions and participation. Doctors often sat at a table (high table) in front of the room where the doctor chairing the meeting sat, and where the patient files were placed. Meanwhile, the rest of the attendees sat in seats arranged in rows, such as in a classroom arrangement, therefore engagement and inclusivity seemed limited [34].

### Audits as a potential avenue for learning

Despite the perceived shortcomings of M&M audits, these audits were also perceived as an avenue for learning from identified gaps in care. Therefore, audit teams often used these meetings to reflect on their actions, by examining selected cases, identifying gaps in care, and developing appropriate measures to improve care.

*We usually do our audits on the spot. And on the spot here means we usually require the people who are on the ground to give us their findings. To give us their interventions and the outcome at which we all learn from it. Then, we are like okay, what was best that was done, what is it that was not done is and what was to be done. Then from there, it becomes a learning point for either of us, those who are on duty and those who are not on duty. (Nurse manager) [hospital Y]*

As a result, positive outcomes linked to reflections and learning points from M&M audits were reported. These include sourcing for essential equipment, such as continuous positive airway pressure therapy (CPAP) machines that were lacking and increasing awareness on essential clinical guidelines which informed early interventions for newly admitted babies. For example, in hospital Y, it was reported that as a result of routinely conducted audits and gathering of evidence, the clinical team had incorporated changes specific to clinical management of newborns into the Ministry of health-specific pediatric protocol that was lacking.

*I think over time like even in the last three years I have been here we have added different data points to be able to change as we recognize something has had a bad outcome, we do the audit, and it is either added to the [Ministry of health pediatric] protocol or added to the card so that we can consistently carry forward improvements. (Pediatrician) [hospital Y]*

Interestingly, M&M meetings also provided a space for intra- and interprofessional interactions, therefore providing an opportunity for strengthening social ties and relationships, which potentially reduces the effects of hierarchy. As a result, all staff irrespective of their seniority are encouraged to speak up about errors and gaps in care, and therefore, promoting learning.

*For example, during audit meetings in hospital Z, tea and snacks were provided to the attendees, during tea-time, we observed health workers chatting and catching up among themselves. In one meeting, one of the NBU medical doctors was exiting the hospital for further studies, we observed that the departmental leader had mobilised the NBU team to buy a farewell present for this team member, which was presented to her at the end of the audit meeting. At this point, her efforts towards improving care delivery and participation in the audit meetings was recognized, after which a cake cutting session to celebrate the milestones was conducted. [Field notes, Hospital Z]*

### The perceived role of leadership in promoting successful audits

We further observed that these positive outcomes and overall learning from case reviews were enabled by dedicated unit and/or departmental leadership. In such instances, leaders made deliberate efforts to engage all healthcare providers attending audit meetings in the review of cases, facilitate identification and discussions of gaps in care, and follow up recommendations from audit meetings. For the latter, this was achieved by either delegating specific action points or engaging the hospital management where action points were needed such as purchasing equipment.

*My experience is, some, most of the time you learn a lot depending on who chairs the meeting. Because when we start auditing mortality, we learn where we went wrong, and we try to learn what to do next time to improve. Some people chair, who can’t even teach or tell you anything or you won’t discuss, it is just the data and that’s all, but mostly they have been educative to me…because you can discuss issues. If it is the paediatrician, he can tell you what is happening or what happened to that baby. What could have happened? If it is the gynaecologist, it is the same. So, kind of it is a learning experience. To try and improve the services. (Nurse) [Hospital X)*

### Role of institutional culture in enabling learning

In addition to the role of leaders in enabling learning during audits, we observed that learning was also enabled by a positive work environment where the conduct of audits was blame-free. To cultivate this environment, all three hospitals had adopted a general approach to the review of cases, by encouraging participation of all providers who were involved in care provision to speak up during the review and illustrating lessons on what could have been done better without blaming or shaming. For instance, in hospital Y, a culture of openness, with minimal victimization, prevailed and we observed how this was enabled by learning-oriented leadership and mission-driven values. This culture appeared to promote active participation and accountability at the individual level, therefore participants openly reflected on cases without fear of being victimized or blamed for the events that might have led to reported poor outcomes.

*During a mortality review of a case of a child who had been referred and managed at the facility by the pediatric surgical team, it was apparent that there was a delay in communication on the status of the patient just after surgery. The patient had been brought to the paediatrics unit, reviewed by a medical officer intern who said during the meeting, “I noted that the baby was in shock, but my mistake was that I did not call anyone”, immediately, a paediatric consultant who was in attendance supported this team member, she acknowledged that the nursing team had done well. She, however, added, “I think the nurse who had assessed the patient and began fluids did well…the failure was on our side, the medical team. [Field notes, M&M audit meeting, hospital Y]*

We also observed that these efforts to promote and create a blame-free environment were not only driven by the desire to promote collective learning and accountability through audits but also by the local politics and fear of litigations. We observed that all meetings in the two public hospitals in our study took a third rather than a first-person narration or a “collective” phrasing such as “we” rather than “I/you”. Healthcare providers, therefore, made attempts to shield individuals, and by extension the hospitals, from consequences that may arise.

*…Specifically, for maternal and neonatal deaths, there is also a lot of politics around it. So, when you come to these meetings, a lot of effort is made to make sure that there is no implication of a specific person, you get, so they try to make it a third-person kind of thing. So, it is discussed in the third person, so for example, if am presenting, it is unlikely I will say unless there is a need to, I could say am the one who saw this baby, and this is what I did. But most of the time it will be “the on-call was called came saw the patient and this is what they did” because sometimes people tend to, this is human nature. Especially where there is like political interference and where you have been told like people are… what we refer in Kiswahili Kuwamulika [expose], so you will try and make sure that you don’t point accusing fingers at individuals… [Medical officer, hospital X]*

Although the review processes generally adopted a victimization-free approach during meetings, it was reported that there were instances where individuals were sometimes reprimanded before or after the meetings. One of the unit managers, however, indicated such a move was more about fostering accountability rather than victimizing individuals. Nonetheless, this compromised the ability of healthcare providers to speak up, own up, and learn from errors.

*…There are instances whereby after the audit and we are done, I will be called back and asked questions because you were with the patient… it will be like a punishment. There are reports that you write, called incidence letters… Ok, …we see them as punishment, but they are not because we just explain what happened, but mostly they seem I’m…I’m wrong and it’s like a punishment. So, some are called back and followed up more. They might even end up being punished. (Nurse) [hospital Y]*

We further observed interprofessional shifting of blame and accountability between some of the healthcare providers in the newborn units. For example, where errors and gaps in care were observed following a case review, there were instances where nurses’ representatives in the meeting felt the observed errors were due to either inaction or delay from the doctors’ end and vice versa. This, in a sense, fostered a culture where each professional in attendance was ready to defend “their own”, in such scenarios interprofessional learning was compromised.

*It…well it would be a tendency for people to first side with their cadre that is human … for example if it was an error in nursing then, of course, the nurse team will side themselves. If it was an error in the clinical side or a decision that was made that did not involve the team members, people will side with that, it's the norm. As much as people are almost shouting at each other, the goal is that a solution is reached and most of the time a solution is reached. (Clinical officer) [hospital Y]*

## Discussion

Our work sought to gain an understanding of how M&M audits could be utilized as a space for learning, improving care delivery processes, and accountability, with a deliberate focus on healthcare workers’ perspectives on audit implementation processes. Our findings suggest that process-related factors such as the experiences of audit team members, relationships and interactions, audit team members’ perceptions on the usefulness of M&M audits, and institutional cultures are equally as important in shaping the outcomes of audit processes as the structural elements are. These findings are further supported by a recent review (2021) [26] which concluded, in addition to the role of structural inputs that have been heavily focused on in the MPDSR implementation (e.g. availing guidelines and training staff), the people-related factors need to be considered. These include their interactions and relationships, motivations, and communication mechanisms, and how these may also influence implementation processes. However, these have not been adequately explored in the existing literature.

Inclusive interprofessional/multidisciplinary meetings with participants reflecting how frontline care is delivered have been identified as a key contributor to the success of M&M audit meetings in achieving positive changes [18, 20]. This is not only limited to attendance but also linked to the call for active participation in open discussions that seek to identify and analyze potentially modifiable factors linked to observed poor outcomes [18, 20]. We observed limited interprofessional participation marked by irregular attendance by the nursing team and “strained” interactions between doctors and nurses during these meetings. Similar to these findings, other studies in Tanzania and Sudan have reported limited participation, with doctors often being the majority of attendees, contributors and consequently influencing audit meeting deliberations and consensus [35, 36]. The studies further attribute such limited participation to professional hierarchies and participants’ characteristics such as fear of public speaking. The negative influence of professional hierarchies on audit processes has been reported by other studies, which suggests that hierarchies stifle participation by more junior staff and nurses [18, 37, 38], undermining collective team performance and learning.

Similarly, our findings suggest that expert power influenced team relationships and interactions, and consequently their participation in audits. The pattern of doctors convening the meetings with junior doctors as the main presenters and contributors reflects pre-existing hierarchies within the medical profession, and overall, medical dominance over other professions [39]. We link this assertion from Steve Luke’s third dimension of power which argues that power can operate at a deeper invisible level, controlling others’ ideologies so that actors may unintentionally follow the dictates of power even against their interests [40]. Therefore, joint collective interprofessional team learning and collective participation that reinforces ownership and realization of positive changes can only be achieved if there is a readiness to dissipate and share power, for example, by having rotational chairs across cadres, which may discourage dominance.

In addition to professional cultures, reported delay or poor implementation of recommendations from M&M audit meetings demotivated health workers from attendance and active participation. While positive outcomes of audits were reported over time, they were still sub-optimal, as demonstrated by the recurrence of issues from meeting to meeting. The observed delay was not unique to our setting. Other studies in Burkina Faso, Tanzania, and Uganda have reported either an observed delay of up to several months, poor implementation, or non-implementation of recommendations from audit meetings [36, 38, 41, 42]. These studies also reported poor attendance and lack of participation in audit meetings because participants were demotivated due to a lack of any positive change from recommendations of the audit meetings. Some of the reasons that have been highlighted as to why recommendations are not followed up and implemented include insufficient: commitment of participants, managerial support, and human and material resources [14, 42].

Implementing audit-generated recommendations may be a complex process involving different stakeholders at various levels of the healthcare delivery system, which could partially explain the reported delays and non-implementation of audit-generated recommendations. However, where positive outcomes were reported, leadership and institutional cultures that enabled positive work environments played a key role in facilitating participation, consensus, uptake of action points, and follow-up to ensure implementation of actions. These were mainly nursing or doctors’ team leads/unit managers who took responsibility to engage the hospital management for their support. In support of our findings, drivers or agents of change were reported as a key factor in the successful implementation and sustaining of a perinatal problem identification programme (PPIP) implemented in South Africa [43]. In this study, a driver or agent of change was considered to be an “interested person who is [the] driver to roll out PPIP & stay[s] motivated to make improvements*”*, and these were identified to be managers and clinicians who were committed and took responsibility for implementing the programme. Therefore, the role of leaders or change agents or champions in quality improvement initiatives such as audit processes is fundamental, as they are enablers of positive changes [44, 45].

Furthermore, existing literature suggests that to attain optimal participation and open discussions that promote learning, a victimization/ blame-free environment must be cultivated [17, 18]. Our findings highlight the efforts of our study hospitals to create a culture that promotes trust and fairness, for instance, using “collective” phrasing in acknowledging human-related errors and gaps in care, such as “we failed” rather than “you failed”, aimed at promoting openness and minimizing individual victimization and blame [46]. These strategies have been suggested to promote speaking up and attaining collective consensus. Despite deliberate efforts to create a blame-free environment, concerns remain over victimization beyond M&M meetings, therefore raising pertinent questions on the balance between creating a blame-free culture and maintaining accountability [14, 17, 36, 38]. This inhibits the creation of a just and trusting culture within hospitals and further indicates that concerns about blame may never be eliminated within audit spaces or clinical work environments.

Finally, it is important to acknowledge that institutional and professional cultures are embedded within a larger social and policy context. The need for clear implementation guidelines for the execution of mortality reviews and an enabling environment with support from governments has been highlighted [17, 47]. WHO recommends the need for legal and ethical frameworks in setting up and conducting mortality audits [8]. This has further been re-emphasized by other studies, suggesting that the absence or existence of legal protection influences the ability of audit team participants to fully take part in audits [12, 48, 49]. We report the influence of political climate on the conduct of M&M meetings, where participants are careful not to be implicated where errors and gaps in care are identified. This suggests that in the wake of increased medical lawsuits, individuals would be unwilling to disclose and discuss adverse events in instances where anonymity for both health workers and clients cannot be fully achieved [8]. This has implications on the potential for learning and improving care delivery processes from mortality audits. Hence need for institutionalizing a legal framework that ensures mortalities are reported and discussed in spaces where accountability and confidentiality of individuals are guaranteed [47].

## Conclusion

In summary, our findings suggest that M&M audit meetings provide a space for meaningful discussions, which may lead to learning and help prevent the recurrence of errors and care gaps that lead to adverse events. However, a lack of participation of all frontline staff engaged in care (including nurses), lack of observed positive outcomes, and negative experiences may reduce their usefulness. Addressing staff disengagement by implementing recommendations from audit meetings requires the commitment of both senior staff and hospital management who may be considered as “agents of change” in this context. Similarly, there is a need to create an enabling environment where hierarchies, negative use of power, and victimization and blame are challenged. This may promote active participation and enhance positive relationships and interactions, therefore promoting open discussions among staff including reporting and speaking up about errors and gaps in care. Consequently, this will help foster learning, improved care processes, and individual and team accountability.

## Supplementary Information

**Additional file 1.** Audit observation guide.

**Additional file 2.** Interview guide.

**Additional file 3.** Audit structure and process illustrative quotes.

## Data Availability

The datasets used and/ or analyzed during this study are available from the authors upon reasonable request and with permission of the KEMRI-Wellcome Trust Research and Governance committee.

## Abbreviations

CPAP: Continuous Positive Airway Pressure therapy
HICs: High-Income Countries
KEMRI: Kenya Medical Research Institute
LMICs: Low and Middle-Income Countries
MPDSR: Maternal and Perinatal Death Surveillance and Response
M&M: Morbidity and Mortality
NBU: Newborn Unit
PPIP: Perinatal Problem Identification Program
SSA: Sub Saharan Africa
WHO: World Health Organization

## Footnotes

1 **Clinical officer**s (**COs**) are non-physician clinicians, they are mid-level healthcare providers who have received less training and have a more restricted scope of practice than physicians. In Kenya, they are regulated by the Clinical Officers Council, who oversee their training, registration and licensing.
