# Supplementary material for: Exploring healthcare workers’ perceptions on the use of morbidity and mortality audits as an avenue for learning and care improvement in Kenyan hospitals’ newborn units
Source: BMC Health Serv Res. 2022 Feb 10;22:172. doi: 10.1186/s12913-022-07572-8 (PMC8832787; doi:10.1186/s12913-022-07572-8)
Supplement: Supplementary file 1 — Additional file 1. Audit observation guide. [file 12913_2022_7572_MOESM1_ESM.docx]

**Non-participant observation guide for audit meetings**

**Research team will observe the following:**

1. **Team composition**

- Is the team diverse? (Age, gender, cadres, hierarchy levels, students)
- What role does each team member play? (Listen to introductions of members, affiliations, qualifications, years of experience).
  - *Explore more about their roles – after meetings/interviews.*
  - *Note* *professionals who have not attended the meeting. Did they give apologies? Is this a pattern?*

**2. Team Leadership**

- Observe the leadership (*What is the role of the facilitator in the hospital? Qualifications?)*
- What methods are used: participatory/structured.
- Does the leadership provide an environment for support of members to freely discuss issues relating to quality and patient safety?
  - *Did all members talk? Who talked and what did they say? Who didn’t talk and what is their role in the hospital?*
  - *What issues did the team focus on? Who raised the issues and why?*
  - *What does the facilitator do to ensure that team members participate?*
  - *Is there evidence of “blame game?”*
- What is the tone/mood/ in the meeting? Observe what the leaders say?
  - *E.g. Are people concerned about the mortality rates? In what ways?*
  - *How are they discussing these issues e.g. discussing by identifying solutions? need to improve service? discussing as a team? Anxious?*

**3. Team goals/Objectives**

- Are the goals of the meeting made clear?
  - *Introductions; mention of the previous meeting?*
  - *Action plans from previous meeting? What they did re: from the previous meeting?*
  - *And, what do they plan to discuss and achieve?*
- At what point are goals explained - at the beginning of the meeting or towards the end?
- How are members reminded of the meeting goals? Who does it? What is their response (Leaflets, ppt, verbal and by whom?)
- Are team members aware of the meeting, venue of the meeting (observe punctuality, space)

**4. Individual competencies**

- How are individual skills and abilities demonstrated during the team process;
  - *Does the Audit facilitator allow different staff within different cadres and roles to contribute their ideas?*
  - *Is there a concern that some staff don’t participate?*
- Are all members given a chance to share views in their different abilities – how?

**5. Team interactions**

- Observe team interactions – how are members interacting – (talking, facial expressions?).
- Do team members make effort to participate in the discussions or are they called upon, encouraged by the team leader?

**6. Trust**

- Do the team leader encourage team members to contribute to the discussions regardless of their role hierarchy?
  - *What do they say. Are nurses able to give examples where a senior doctor compromised practice or either way.*
- Do all team members express their thoughts and opinions without fear? (note who is saying what and responding to whom (Junior vs senior staff, within senior staff and within junior staff). How are they communicating/discussing these issues -gauge the tone, facial expressions, pauses etc)?

**7. Professionalism**

- How are the team members making decisions? Are they involving the views of all participants? Are they respectfully listening to teach other?
- Are their discussions guided by existing national or international guidelines?
  - *Are maternal and perinatal audits integrated based on WHO’s recommendations? Are they aware of this framework? Look for patterns in all the hospitals.*
- How are ideas exchanged between and within different professional cadres? Observe how difference of opinion is being resolved. Observe if team members give any feedback and how this is received by other members.

**8. Reflection**

- What techniques is the team using to solve problems, discuss patient safety and quality issues.
- *How they discuss mortality cases? What went wrong in those cases? Can they attribute a cause to death? How do they do this: as a team or individuals? Can they identify action plan – and how do they do this, do they write minutes? do they assign people to follow up on the action plan? How do they plan to achieve the action plan (training, individual conversations, leaflets)?* Is there sufficient documentation in the chart (ie is the chart of the patient physically present?) When timing of events is described, do they refer to the chart or patient file?
- Are members given a chance to reflect on each case discussed. If so, how is this done? Observe what factors they are reflecting on -context? patient safety? Errors? Staffing? Resources?
- Are they reflecting as individuals or as a team?
- How is team reflection translating into decisions/ benchmarks? Who is taking the lead – and what is their role?

**9 Agency**

- Observe if there are differential power issues between and within staff cadres?
  - *Interactions, who makes decisions, who talks most? Are decision agreed by all members, are some staff interrupted when they are trying to talk?*

**10 Context**

- Observe resources available for use by health care and audit teams; space, basic materials such as notebooks, pens, tea?
- Is anyone from the administration in attendance?
- Do the audit team have adequate time to attend the meeting? Or could they be attending to a patient or an administrative duty concurrently?
  - *Do all participants sit in the meeting throughout or do they leave to attend to other chores. Do we have other professionals coming in late, when the meeting has already started?*
