# Supplementary material for: Exploring healthcare workers’ perceptions on the use of morbidity and mortality audits as an avenue for learning and care improvement in Kenyan hospitals’ newborn units
Source: BMC Health Serv Res. 2022 Feb 10;22:172. doi: 10.1186/s12913-022-07572-8 (PMC8832787; doi:10.1186/s12913-022-07572-8)
Supplement: Supplementary file 2 — Additional file 2. Interview guide. [file 12913_2022_7572_MOESM2_ESM.docx]

**Interview guide**

**Closed ended questions**

| **Interviewee Code** |  |
| --- | --- |
| **Hospital** |  |
| **Position** |  |
| **Age** |  |
| **Gender** |  |
| **Work Experience (yrs)** |  |
| **Experience in NBU (yrs)** |  |

**Open- Ended Questions**

**Could you tell me about the team you work with?**

<<*Who do you consider to be part of your team? Why do you say so?* (*Explore their perception within own cadre and with other cadres)>>*

**Let’s talk about your (cadre specific) team, how do you relate with each other? *Then*, how do you relate with other cadres that you work with?**

*<< how do you communicate among each other? step into shift allocations, preferences to work with specific individuals, explore differences in education/level of experience and how it influences relationships. Explore Inter-cadre relationships, differences, “wars”>>*

**What mechanisms are in place to promote interactions and team working among the different cadres? What has worked so far? And what are the challenges that impede team working?**

**Are there any shared objectives or goals in place that you as a team work towards? Probe for examples, how such are set and whether there are efforts to evaluate progress.**

**How would you describe your team leadership? (Could be a nurse manager, consultant or head of department or an opinion leader (not positional)**

*<< As a nurse/doctor/nutritionist, who is your team leader? Why do you say that? How do you relate with your team leader? What is working well? What is not working well? What could be done to make things better?*>>

**In your view, does the leadership style here promote team working? In what ways?**

*<< For TLs; what approaches do you use to promote cohesion and team working among your team? What has been your experience with this?>>*

**How do the team leaders of various cadres relate with each other?**

**Do you hold any cadre specific departmental meetings? What about inter-cadre departmental meetings?**

*<<How often? How do people interact during such meetings? What kind of issues are usually raised? How are such issues addressed? And by who? Are such meetings useful?>>*

I would like to understand some processes that I have observed in your ward, I will ask you more about how each is conducted? who is involved? with what? And why?

**Could you please describe to me how a HANDOVER is done?**

*<<What would you say are the key procedures during handover? Why are they important? Who is part of the handover and why? What information do you usually handover? Guidelines? Perceptions on process? What would you like changed/improved?>>*

**Could you please describe to me what an ADMISSION PROCESS entails?**

*<< Who is involved? How do NBU staff interact with other unit staff during admission? Information handed over? Guidelines? Do errors happen during admission? How are they identified and discussed?* >>

**Could you please describe to what a WARD ROUND entails?**

*<<Who is involved? Who leads? Communication between teams involved? Why are rounds important? Probe for learning, identification and discussion of errors>> >>*

**During ward rounds/handovers/admission/meetings are there opportunities to talk about errors or gaps in care? How are these errors identified? How are they discussed? Are suggestions for improvement made? How are they acted on?**

<<Could you give an example? Probe for management approach in dealing with errors>>>

*We will now move on to talk about mortality audits;*

**Can you please describe to me how a mortality audit is planned for?**

*<< Who organises this meeting? Is the venue and agenda of the meeting communicated? If yes, how? By who? When? Who is invited? Is this information communicated to you early enough to make time to attend? >>*

**How is the meeting conducted (prompts from observations on the audit process)?**

*<<How do you select cases for discussion? What guides the team while reviewing a mortality?>>*

**What has been your experience of being a member of a mortality audit team?**

*<<Step into team leadership,* *issues around professionalism, trust and agency* *as observed in the audit teams e.g. ease/difficulties of information sharing (such as talking about omissions in care or speaking up against unprofessional behaviour)>>*

**Are there any guidelines that guides the conduct and outcomes of mortality meetings?**

Do you receive any support from the hospital and hospital administrators to conduct mortality audits? *(If yes, step into the type and nature of organisational support)*

**How is the information or findings of a mortality audit used?**

*<<Do you think mortality audit is important? Why? Step into their perceived value of mortality audit meetings.>>*

**What are the positive changes you’ve seen because of conducting mortality audits over time***? (What is working well, what is not working well and what would you like to see different/change in future?)*

Is there anything else you would wish to add on mortality audits and mortality audits teams more generally?
