# Supplementary material for: Exploring healthcare workers’ perceptions on the use of morbidity and mortality audits as an avenue for learning and care improvement in Kenyan hospitals’ newborn units
Source: BMC Health Serv Res. 2022 Feb 10;22:172. doi: 10.1186/s12913-022-07572-8 (PMC8832787; doi:10.1186/s12913-022-07572-8)
Supplement: Supplementary file 3 — Additional file 3. Audit structure and process illustrative quotes. [file 12913_2022_7572_MOESM3_ESM.docx]

| **Participation**  *You see initially those audits they were tagged to be the clinicians… at a point for them [doctors] it used to be compulsory to attend…you know the way you find things done it used to be the clinicians who used to attend and the team leader of the unit. And probably the nurse can get another extra person who is on duty to attend It was not a meeting that was compulsory for the nurses. [Nurse, hospital Z]*  **Frequency**  *…Every first Thursday of every month it’s all the nurses, clinical officers, and consultants doing nursery audit. I mean we look at any morbidities and mortalities for the month and any big themes [emerging issues] altogether and then any time there is an event of maternal death then we meet as the obstetrics and paediatrics department together to review that within 24 hours. [Paediatrician, hospital Y]*  **Case review Process**  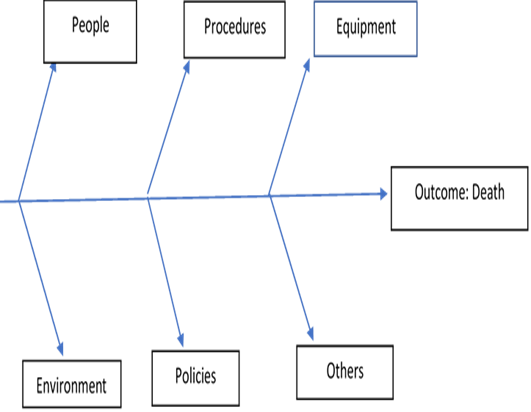  Figure 1: Use of fishbone diagram for death reviews (from nonparticipant observation of the M&M meetings)  *That is the other thing that needs a bit of improvement because our format in terms of the audit, there are some pediatric, actually neonatal forms that were developed recently which ideally, we should be using. An intern can quickly go through the file and be able to put all the information that is needed, so that you are able to grade in terms of where the errors occurred, is where, is it at home? Was a delay in transfer or is it a system failure within us, in terms of now the care? Those forms are there maybe we have not reached a point where we are using them as they should be… [Paediatrician, hospital X]*  *During this audit meeting, one of the medical officers was presenting summary statistics for the month, as often was the tradition in facility Z. As he presented a table on temperature taking, it was noted that taking temperatures at admission was still an issue (this had been discussed in two previous audit meetings we had attended, and a consensus had been reached on the need for temperature taking on admission). The nursing officer in charge, then responded to this finding, saying, “I have sampled files, it is not done, the issue is lack of thermometers, but I have done a requisition of thermometers for all clinicians and nurses”. While lack of thermometers had been highlighted in the previous meetings, we observed that almost 3 months later, it hadn’t been addressed, no immediate action taken nor subsequent management in care addressed, despite frequent discussions in the audit meetings. [Field notes, M&M audit meeting, hospital Z]* |
| --- |
